# Does health and social care provision for the community dwelling older population help to reduce unplanned secondary care, support timely discharge and improve patient well-being? A mixed method meta-review of systematic reviews

**DOI:** 10.12688/f1000research.25277.1

**Published:** 2020-07-31

**Authors:** Shoba Dawson, Patience Kunonga, Fiona Beyer, Gemma Spiers, Matthew Booker, Ruth McDonald, Ailsa Cameron, Dawn Craig, Barbara Hanratty, Chris Salisbury, Alyson Huntley

**Affiliations:** 1 Centre for Academic Primary Care, Bristol Medical School, Population Health Sciences, University of Bristol, Bristol, UK; 2 Population Health Sciences Institute, Faculty of Medical Sciences, Newcastle University, UK, Newcastle, UK; 3 Alliance Manchester Business School, University of Manchester, Manchester, UK; 4 School for Policy Studies, University of Bristol, Bristol, UK; 5 Population Health Sciences Institute, Biomedical Research Building, Campus for Ageing and Vitality, Newcastle University, UK, Newcastle, UK

**Keywords:** meta-review, systematic reviews, health care, social care, community-dwelling older population, unplanned admissions, patient well-being

## Abstract

**Background: **This study aimed to identify and examine systematic review evidence of health and social care interventions for the community-dwelling older population regarding unplanned hospital admissions, timely hospital discharge and patient well-being.

**Methods: **A meta-review was conducted using Joanna Briggs and PRISMA guidance. A search strategy was developed: eight bibliographic medical and social science databases were searched, and references of included studies checked. Searches were restricted to OECD countries and to systematic reviews published between January 2013–March 2018. Data extraction and quality appraisal was undertaken by one reviewer with a random sample screened independently by two others.

**Results: **Searches retrieved 21,233 records; using data mining techniques, we identified 8,720 reviews. Following title and abstract and full-paper screening, 71 systematic reviews were included: 62 quantitative, seven qualitative and two mixed methods reviews. There were 52 reviews concerned with healthcare interventions and 19 reviews concerned with social care interventions. This meta-review summarises the evidence and evidence gaps of nine broad types of health and social care interventions. It scrutinises the presence of research in combined health and social care provision, finding it lacking in both definition and detail given. This meta-review debates the overlap of some of the person-centred support provided by community health and social care provision. Research recommendations have been generated by this process for both primary and secondary research. Finally, it proposes that research recommendations can be delivered on an ongoing basis if meta-reviews are conducted as living systematic reviews.

**Conclusions: **This meta-review provides evidence of the effect of health and social care interventions for the community-dwelling older population and identification of evidence gaps. It highlights the lack of evidence for combined health and social care interventions and for the impact of social care interventions on health care outcomes.

**Registration:** PROSPERO ID CRD42018087534; registered on 15 March 2018.

## Introduction

In a recent government report on the UK population, it is predicted that in 50 years’ time there will be an extra 8.2 million people aged 65 years and above in the UK. This cohort will comprise over a quarter of the total UK population and equates to the current size of London ^1^. We are already aware of the impact of the ageing population on health care services. In 2017, 3.5 million (22.2%) of total hospital admissions in England were in the 75 years and over age group. Once in hospital, the same population spent 10.7 million days as inpatients if they were not in the last year of life and 7.5 million days if they were in their last year of life ^2^. Once in hospital, there are further issues that need to be considered. Longer, potentially unnecessary hospital stays (delayed discharge) are likely to have a detrimental effect on the older population. NHS Improvement reports that 35% of 70-year-old inpatients experience a decline in function compared to preadmission, and this rises to 65% for people over the age of 90 ^3^.

Discharge care for the older population often involves ongoing health care and may also involve social care provision. Social care provision is a balance for local authorities between protecting the most vulnerable in society and the resources available. Insufficient numbers of care home staff and affordable care home places can result in older people having an inappropriately extended hospital stay ^4^. The National Audit office estimate that the number of older inpatients no longer requiring acute care but still in hospital is around 2.7 timers higher than official statistics suggest ^5^. The lack of integration of the health and social care sectors is historical as well as financial and how to improve the situation has been a challenging and controversial debate for decades. In 2014, The Barker Commission concluded that the support needed by older people to navigate through the existing health and social care system needed to be simplified, with services built around people’s individual needs. It further concluded that integration of health and social care provision can only happen if traditional definitions and divides are broken down ^6^.

In 2017, NHS England set out an ambition to make a national, comprehensive move to integrated health and social care by the development of sustainability and transformation partnerships (STPs) ^7^. These STPs are local partnerships of NHS and local authority organisations with the aim of taking more control of local funding and services to improve the health and wellbeing of the population ^8^.

The overall aim of this meta-review is to provide an evidence base of efficacy for health care, social care and combined health and social care interventions for the older population in terms of the impact on hospital admissions, timely discharge and patients’ quality of life.

**The aims of this meta-review were:**

A) To identify effective interventions to deliver health and social care to the community-dwelling older population.
B) To understand what is important to the community dwelling older population concerning their care, and important to the professionals providing it, with respect to unplanned hospital admissions, inpatient stays and patient wellbeing.
C) To identify definitions of social care and combined health and social care for older people that have been used in the systematic review literature.
D) To identify the components of the health and social care interventions that potentially complement and reduce unplanned hospital admissions, support timely appropriate hospital discharge and enhance well-being.
E) To identify future mixed-methods synthesis by matching intervention effectiveness with related patient experience to facilitate suggestions for effectiveness or ineffectiveness of intervention approaches and produce research recommendations.

## Methods

### Search strategy

We were guided by the Joanna Briggs methodology for Joanna Briggs Institute umbrella reviews ^9^ and reported according to PRISMA guidance ^10^. A search strategy was developed in collaboration with the research team with the advice of an experienced information specialist (FB). It combined the following concepts: ([Health care terms or social care terms] AND [overview of reviews filter]) ^11^ NOT [LMIC (lower- and middle-income countries) OR children] ( *Extended data*, Appendix 1 ^12^). It is more robust to exclude LMIC countries than to select studies where higher income countries are indexed, since indexing terms for higher income countries are not always added. The search did not specify ‘older people’ because scoping revealed that not all relevant studies were indexed in this way.

Thesaurus headings for each concept were combined with terms in the title and abstract fields and translated as appropriate for each database. All search results were downloaded to Endnote and de-duplicated. The following databases were searched: The Cochrane Library (Wiley); MEDLINE (OVID); Embase (OVID); Cumulative Index to Nursing and Allied Health Literature (CINAHL) (EBSCO); PsycINFO (OVID); Social Sciences Citation Index (Web of Knowledge); Conference Proceedings Citation Index - Social Science & Humanities (Web of Knowledge); International Bibliography of the Social Sciences (IBSS) (ProQuest); Social Services Abstracts (ProQuest); Social Care Online.

Searches were restricted to Organisation for Economic Co-operation and Development (OECD) countries and to the past five years (January 2013– March 2018). A 2013 limit ensured capturing data from at least previous 30–40 years, as determined by examining relevant 2009/2013 systematic reviews for their search limits. Thus, any studies on recent changes to care provision such as the GP Contract Changes of 2004, the introduction of four-hour wait targets in emergency departments in 2004 and the Health & Social Care Act 2008 were included. There were no language restrictions, provided an English language abstract was available for initial screening. Forward and backward referencing was conducted on all included full systematic review papers to identify any further relevant systematic reviews. Detailed methods can be found in our PROSPERO registration protocol CRD42018087534.

### Types of study

We sought the highest-level evidence available for our research questions. In our health care question, we expected that to be systematic reviews of randomised controlled and controlled trials. In our social care and combined health and social care questions we expected systematic reviews of controlled/observational studies. We used the DARE guidance to assess whether a review can be classified as a systematic review ^13^.

### Population

Community-dwelling older (≥65 years) people. If systematic reviews included data from participants older and younger than 65 years of age, we only included the systematic reviews if the older adult studies were presented separately and formed at least 50% of the included studies. Community dwelling was defined as all residential living including domestic, care, and nursing and sheltered (extra care) housing. Reviews that solely focus on carers or patients in end-of-life care were excluded, as this is beyond the scope of the current review.

### Interventions

Health care interventions were those received by a community-dwelling older population that did not involve include an admission into a secondary or tertiary care hospital.

Social care: as defined in the Care Act 2014:

"Adult social care" -

a) includes all forms of personal care and other practical assistance for individuals who by reasons of age, illness, disability, pregnancy, childbirth, dependence on alcohol or drugs or any similar circumstances, are in need of such care or other assistance, but

b) does not include anything provided by an establishment or agency for which Her Majesty’s Chief Inspector of Education, Children’s Services and Skills is the registration authority under section 5 of the Care Standards Act of 2000" ^14^.

Combined health and social care: any combination of the above, whether provided by separate services but in a co-ordinated way, or through fully integrated services.

### Comparators

For the quantitative reviews, any comparator was suitable in the studies described in the included systematic reviews. For the qualitative reviews this was not applicable.

### Main outcomes

Outcomes for quantitative systematic reviews were: 1) unplanned hospital admissions/readmissions, 2) length of stay (LOS) and 3) patient well-being.

We were inclusive in our use of patient well-being outcomes and, in addition to measures of the quality of life, we included measures of social isolation and loneliness. We included systematic reviews of qualitative data if they described: patients’ or health and social care professionals’ experiences of healthcare or social care or combined health and social care relevant to unplanned hospital (re)admission or timely discharge and/ or quality of life.

### Data extraction

Titles and abstracts were independently assessed for inclusion by two reviewers using pre-defined criteria as detailed above. Disagreements were resolved through discussion and, where necessary, consultation with a third reviewer. For publications of potential relevance, full papers were assessed independently by two reviewers with disagreements resolved as above. Data extraction was undertake using a customised spreadsheet ( *Extended data*, Appendix 2 ^12^) in Microsoft Excel by one reviewer (SD) with a random sample of 20% independently screened by two other authors.

### Data analysis, risk of bias assessment and synthesis approach

We were guided by the Joanna Briggs methodology for umbrella reviews ^9^. All data analysis was predominantly descriptive in nature. Where data synthesis was performed it comprised of narrative groupings of data or ideas.

Descriptive analysis of evidence

We narratively presented the aims, specific intervention definition, outcome measured and authors conclusions from both quantitative and qualitative reviews in both text and tables grouped by overall intervention type. If there were more than one systematic review of the same or similar data, we have reported any differences.

Risk of bias (quality) assessment

Quality appraisal of the systematic reviews of quantitative data was conducted using the ROBIS tool ^15^. This comprises four main domains assessed by signalling questions: eligibility, identification of studies, collection/appraisal of data, and synthesis and findings. Quality appraisal of systematic reviews of qualitative data and mixed methods reviews was conducted using the GRADE-CERQual tool ^16^. This approach includes four components for assessing how much confidence to place on the findings: the methodological limitations of the individual qualitative studies contributing to the review finding; the relevance to the review question of the individual studies contributing to the review finding; the coherence of the review finding; and the adequacy of data supporting a review finding.

Quality appraisal was conducted independently by two reviewers for a 10% sample of included systematic reviews. There was a high level of agreement between the reviewers (94%), therefore the remaining reviews were appraised by one reviewer (SD) and checked by a second reviewer (AH). Any discrepancies were resolved through consensus. We did not exclude on quality but took account of the quality of evidence when discussing the findings of the included systematic reviews.

Evidence map (addressing Aims A & B)

An evidence map was produced detailing volume of evidence (number of reviews) per intervention per outcome measure and showing gaps in evidence. 

Evidence summary (addressing Aims A & B)

An evidence summary was produced for interventions which had a positive or no impact on our outcomes of interest. Positive or no impact were defined as any stated by the authors’ results and conclusions at systematic review level. The majority of these were based on evidence from A) *meta-analysis* of randomised controlled trial data; B) *narrative evidence* predominantly from RCT data, but no meta-analysis performed, C) *limited evidence* means two or less RCTs, D) *low quality evidence* means predominantly non-RCT evidence. A small number of qualitative reviews were identified and where present are denoted by Q.

Definitions of combined health and social care (addressing Aim C)

If present these were collated in one table and discussed within the results and discussion section.

Complementary components of health and social care interventions (Addressing Aim D).

These were described in the results and the discussion sections.

Future mixed methods synthesis (addressing Aim E).

We identified existing and future mixed methods synthesis of evidence to facilitate suggestions for future research.

## Results

### Overview

There were 71 systematic reviews included in this meta-review: 62 quantitative reviews, seven qualitative reviews and two mixed methods reviews ( *Extended data*, Appendix 3: PRISMA diagram ^12^) 52 reviews were concerned with health care interventions, of which 46 were quantitative reviews and six were qualitative reviews. 19 reviews were concerned with social care interventions of which 16 were quantitative, two were mixed methods and one qualitative review. *Extended data*, Table 1 ^12^ contains a description of the included reviews.

***Patient populations*.** Systematic reviews of health care interventions focused on the older population (17), chronic obstructive pulmonary disease (COPD) (12), heart failure/atrial fibrillation (10) with a smaller number of reviews on stroke (2), dementia (1), Parkinson’s (1), vertebral compression fractures (1) and mixed chronic conditions (2). In addition, there were six composite reviews covering broader topic areas. Systematic reviews of social care included the older population (16) and dementia (3).

***Types of interventions*.** Health care interventions were organised into three groups: *Care in the community* (Reviews 1–35) ^17– 51^, *Urgent care at the community/hospital interface* (Reviews 36–39) ^52– 55^ and *Discharge and transitional care at the hospital/community interface* (Reviews 2, 40–52) ^18, 56– 68^. Social care interventions were organised into two groups: *formal social care* (Reviews 53–57) ^69– 73^ and *synthetic social support* (Reviews 58–71) ^74– 87^.

### Risk of bias in reviews

***Health care interventions*.** Of the 46 quantitative reviews of health care interventions, 28 (61%) were determined to be at low risk of bias across all four domains, 13 (28%) had a least one domain determined to be unclear (due to lack of information), and five (11%) had at least one domain (predominantly domains 2 and 3) determined to be at high risk (see *Extended data*, Table 2a ^12^).

There were six qualitative reviews concerned with health care interventions and of these, five were determined to be at low concern across all four domains and high confidence overall. The remaining one review was determined to be of moderate concern over domains two and three and therefore of moderate confidence.

***Social care interventions*.** Of the 16 quantitative reviews of social care interventions, eight (50%) were determined to be at low risk of bias across all four domains, four (25%) had one domain determined to be unclear (mostly domain 3) and four (25%) were determined to have at least one domain at high risk (mostly domain 3 and 4) (see *Extended data*, Table 2b ^12^).

There were one qualitative and two mixed methods reviews concerned with social care interventions and of these, one was determined to be at low concern across all four domains and high confidence. The other review was determined to be a mixture of moderate and low concern and of moderate confidence.

### Addressing Aims, A and B: Effectiveness and peoples’ experiences of health and social care interventions

In this section, the following are summarised: 1) evidence and evidence gaps for health and social care interventions per condition and outcome, and 2) the effectiveness of interventions and people’s experiences of them. Finally, detailed descriptions of the included systematic reviews are given.

### 1. Evidence and evidence gaps by intervention type and outcome

The majority of included health care systematic reviews measured hospital admissions as an outcome (33/52). Two formal social care reviews measured hospital admissions (2/5). No synthetic social support reviews measured hospital admissions (0/14) ( *Extended data*, Table 3 shows an evidence map ^12^).

Timely discharge was only measured in the discharge care intervention reviews (2/14) and was not present in any social care review (0/19).

Within health care systematic reviews, quality of life was measured in 20/52 reviews and was most prevalent in the care in the community intervention reviews, particularly self-management, exercise/rehabilitation and medication review (17/35), as opposed to 0/4 in the urgent care reviews and 3/14 in the discharge care reviews. Quality of life was measured in 17/19 social care intervention reviews of which 10 were focused on social isolation and loneliness.

Only 7/71 of the included systematic reviews were of qualitative data (people’s experiences) and 2/71 contained a combination of quantitative and qualitative data with similar representation across health (6/52) and social care (3/19) reviews.

### 2. Evidence summary by condition and outcome

### Hospital admissions

***Positive benefit*.** The evidence for positive benefit of interventions in reducing hospital admissions is derived from the health care evidence across community, urgent and discharge care interventions focusing on the older population, COPD and heart failure patients ( *Extended data*, Table 4 provides a summary of the evidence ^12^).

In the older population there is meta-analysis-level evidence for positive benefit for both discharge/transitional care for all and influenza vaccination for nursing home residents. For COPD patients there is meta-analysis level evidence for positive benefit for rehabilitation/post-rehabilitation support, influenza vaccination, discharge/transitional care and hospital at home (in place of admission). For heart failure patients there is meta-analysis level evidence for positive benefit for discharge/ transitional care and hospital-initiated case management.

***No benefit*.** There is meta-analysis level evidence for no benefit in terms of hospital admissions for the following health interventions. For the general population, community case management, medication review and nurse-led geriatric ED care confers no benefit. For COPD patients, self-management intervention confers no benefit and for heart failure patients supervised exercise and community case management confers no benefit.

### Timely discharge

***Positive benefit*.** There is meta-analysis level evidence for positive benefit of hospital-initiated case management for heart failure patients.

***No benefit*.** There is no meta-analysis level evidence for any health or social care intervention in any population supporting no benefit for timely discharge.

### Quality of life

***Positive benefit*.** The evidence for positive benefit of interventions in quality of life is present in both health and social care interventions with meta-analysis level evidence for the older population, COPD, heart failure, stroke and dementia patients. For the older population, this includes self-management and reablement interventions; for COPD patients, this includes breathing techniques, Tai Chi and hospital at home (in place of admission) interventions; for heart failure patients, this includes general exercise, Tai Chi, hospital at home (in place of admission), discharge and transitional care; for stroke patients this includes self-management.

***No benefit*.** There is meta-analysis level evidence that supports no benefit for quality of life for the older population with medication review and for COPD patients with post rehabilitation support.

### Peoples’ experiences (qualitative outcomes)

There are nine included systematic reviews of qualitative evidence or a combination of quantitative and qualitative data (mixed methods) evidence: self-management and case management for heart failure patients, self-management for stroke patients, transitional and discharge interventions for older people, rehabilitation and exercise for COPD patients, formal social care (mixed methods) for the older population and reablement (mixed methods) for the older population, These are described in *Extended data*, Table 1 ^12^) and discussed in more detailed in aim E.

### Addressing Aims C and D: definitions of social care and combined health and social care and identifying complementary components of health and social care interventions

***Definitions*.** Definitions of specific social care interventions are listed in *Extended data*, Appendix 4 ^12^ and include reablement, personal support and synthetic social support.

Definitions of combined health and social care were poorly described in the included systematic reviews ( *Extended data*, Appendix 4 ^12^). All 13 definitions identified came from the health care systematic reviews with eight describing care in the community interventions and five describing discharge/transitional interventions. Combined health and social activity were implied as opposed to specifically mentioning integrated working in all these definitions with the exception of Review 41 ^57^. This review included discharge and transitional interventions defined as ‘interventions that could be implemented in any health or social care setting (primary, secondary or community care), as long as they crossed the boundary between two or more settings. The community setting encompassed care given in the community, in patient homes or by social care professionals.’

***Complementary components of health and social care interventions*.** Aim D was to identify complementary components of care across health and social care interventions. By examining the definitions of individual health and social care interventions it is clear to see some overlap of components for the social care interventions reablement and personal assistance with aspects of health professional-driven interventions of self -management, case management, discharge/transitional care and rehabilitation ( *Extended data*, Appendix 4 ^12^). These interventions included a home-based, tailored/individualized (patient-centered) approach, one to one, face to face, co-ordination of integrated care to support people to live their lives as well as possible for as long as needed.

### Addressing Aim E: Future mixed methods synthesis

Mixed methods synthesis that matched evidence of intervention effectiveness with related patient experience evidence were identified ( *Extended data*, Table 5 ^12^). This was limited because only seven systematic reviews with qualitative data ^25, 27, 29, 43, 61, 62, 70^ and two mixed methods reviews ^78, 85^ were included in this meta review.

Two systematic reviews of qualitative evidence were conducted as part of segregated mixed methods reviews. Stroke and self-management (Reviews 10 & 11) ^26, 27^ was conducted by the same authors as was heart failure and case management (Reviews 26 & 27) ^42, 43^. In the latter case, the qualitative synthesis sought to understand the impact of case management on hospital admissions ^42^ for patients with heart failure in a qualitative synthesis ^43^.

Two of the systematic reviews were conducted as integrated mixed methods reviews examining social care for the older person and reablement for the older person ^69, 71^. Social care for the older person is also evaluated in one systematic review of qualitative data (Review 54) ^70^ as is reablement with a systematic review of quantitative data (Review 56) ^72^.

There are two future mixed methods synthesis identified by the meta-review that could be conducted: 1) Rehabilitation and post rehabilitation support for COPD patients and 2) Discharge/transitional care for the older population 3) Rehabilitation and post rehabilitation support for patients with COPD is evaluated in three effectiveness systematic reviews (Reviews 12, 14, 15) ^28, 30, 31^ with a complementary systematic review of qualitative data in Review 13 ^29^. Discharge and transitional care for the older person is evaluated in four effectiveness reviews (Reviews 40-43) ^56– 59^ with two complementary systematic reviews of qualitative data in Reviews 45 and 46 ^61, 62^.

## Discussion

This meta-review describes and evaluates health and social care provision aiming to reduce unplanned secondary care, support timely discharge, and improve patient well-being for the community dwelling older population.

There were 71 systematic reviews included: 62 quantitative reviews, seven qualitative reviews and two mixed methods reviews. Of these, 52 reviews were concerned with health care interventions, and 19 reviews were concerned with social care interventions. There was very little content of these included reviews providing evidence for combined health and social care for the older person which reflects traditional mindsets in practice and research both in the UK and elsewhere ^8^. The reviews included health care interventions targeting the older population as well as specific patient populations such as COPD, heart failure and dementia patients. The reviews of social care interventions predominantly included the older population with some of the studies within the reviews, including younger populations. There were fewer reviews of social care than health care, and within these reviews, there were fewer RCTs than in the health care reviews. Quality appraisal of the health and social care reviews shows that a greater proportion of the health care reviews compared to social care reviews were of a higher methodological standard. This was expected, as systematic review methodology is less common in social care research ^88^. However, it is of note that 50% of the social care reviews were determined to be of low risk of bias overall.

This meta-review maps out intervention research by population and outcome, highlighting evidence but also identifies evidence gaps. Some of these evidence gaps are not of value to pursue; for example, we would not expect most community and urgent care interventions to improve timely discharge. It is unlikely that synthetic social support would impact on hospital admissions.

However, there is an interesting dichotomy in intervention research and measurement in quality of life outcomes; whilst it is a widespread and obvious outcome for self-management, exercise and rehabilitation interventions, it is less prevalent in more medical care provision such as case management, urgent care, discharge and transitional interventions. Patient involvement and satisfaction with care is not only important for patients’ quality of life but also can impact the success of an intervention ^89^. The qualitative data exploring patient and health professional experience when available, can also provide unique insight regarding a specific intervention ^90^.

Social care and synthetic social support evidence in this meta-review tends to focus on subjective outcomes and yet it could be of value for social care provision research to measure more objective outcomes such as the use of care services. The evidence for effectiveness in this meta-review is dominated by hospital admissions and quality of life. Timely discharge is more likely to be the focus of hospital-based care. Across the 71 included systematic reviews, we only identified seven relevant reviews of qualitative evidence and two reviews of mixed-methods evidence. Those interventions measuring hospital admissions were health care focused, and the included systematic reviews were generally supported by a meta-analysis of RCTs. Quality of life outcomes was present in both health and social care systematic reviews; notably, there was no meta-analysis level evidence in social care or synthetic social support interventions reviews. Social care data comprised few RCTs in combination with less rigorous study types of a heterogeneous group of interventions.

A total of 13 reviews focused on combined or integrated health and social care interventions, with only one systematic review having a clear, integrated health and social care definition for its included studies ^57^. Despite the fact we know that health care works with social care within interventions described in the many of the included systematic reviews of health interventions, e.g. ED, discharge and transitional care interventions, there is little mention of social care involvement in these health research publications ^91^. Although we are looking at systematic review-level evidence, this likely reflects the individual study intervention descriptions. This reinforces the view that we persist in researching health care and social care as separate entities and are not acknowledging the combined activities that already exist. Hopefully, we are on the cusp of change with combined health and social care funding coming in to place ^92^. It is also important to acknowledge that more pragmatic evaluation work of health and social care at a local level may be addressing this better.

Finally, we identified mixed methods evidence synthesis within the meta-review. This aim had two objectives. Firstly, to demonstrate the utility of mixed method synthesis in this topic area. For example, the evidence for case management for heart failure both examines effectiveness data for potential reduction of hospital admissions as well as qualitative data exploring patients’ (and health professionals’) experiences of case management ^42, 43^. Secondly, to identify future mixed-method synthesis, i.e. research recommendations for future work. Several topic areas were identified across both health and social care ( *Extended data*, Table 5 ^12^) with notable examples being the use of quantitative (effectiveness) and qualitative (patient experience) in discharge and transitional care ^56– 59, 61, 62^.

There are limitations to conducting a meta-review on such a broad topic area. This meta-review included systematic reviews published in the past five years. This means if a topic area had been reviewed with a definitive outcome prior to this, the evidence does not appear in this meta-review. Some interventions may not have enough primary RCTs or studies generally to warrant a systematic review, e.g. diuretic management of heart failure patients in the community.

A meta-review can only really express a direction of effect of evidence for an intervention type, and the detail is needed to get the full picture. An example of this is the evidence for nurses and geriatricians working in emergency departments. At meta-review level, the evidence suggests that nurses do not impact on patient admission, but geriatricians do. At the study level, we can see that the nurse studies are of high quality and component analysis suggests that the impact depends on the type of nurse and the context ^53^. The geriatrician data comprises lower quality observational studies and therefore needs replication with RCTs ^55^.

The searches for this meta-review were conducted in 2018, reflecting the length of time needed to synthesise such a large volume of evidence. The fact that the meta-review has included systematic reviews and not primary studies give us confidence that the overall evidence base of the interventions is unlikely to have significantly changed. However, this topic area is an excellent candidate for becoming a living systematic review to continue to inform future primary and secondary studies ^92, 93^.

In conclusion, this meta-review of health and social care interventions for the older population provides evidence about the overall effect and the evidence gaps of the included interventions, with qualitative data for selected topics. It also highlights the lack of evidence, detail and discussion for combined health and social care and the lack of high-quality evidence for the impact of social care interventions on care provision outcomes. This meta-review will support future research in health and social care in the ageing population.

## Data availability

### Underlying data

All data underlying the results are available as part of the article and no additional source data are required.

### Extended data

Figshare: Does health and social care provision for the community dwelling older population help to reduce unplanned secondary care, support timely discharge and improve patient well-being? A mixed method meta-review of systematic reviews. https://doi.org/10.6084/m9.figshare.12688487.v1 ^12^.

This project contains the following extended data:

- Table 1- Health and social care interventions systematic review-aims, outcomes and conclusions.
- Table 2- Risk of bias of included studies.
- Table 3- Evidence map of health and social care interventions of included systematic reviews based on conditions and outcomes.
- Table 4- Evidence summary at systematic review level for efficacy for health and social care interventions by outcome.
- Table 5- Mixed methods evidence identified.
- Appendix 1- Example search strategy.
- Appendix 2- Example data extraction form.
- Appendix 3- PRISMA Flowchart.
- Appendix 4- Definitions of interventions.

### Reporting guidelines

Figshare: PRISMA checklist (Appendix 5) for ‘Does health and social care provision for the community dwelling older population help to reduce unplanned secondary care, support timely discharge and improve patient well-being? A mixed method meta-review of systematic reviews’. https://doi.org/10.6084/m9.figshare.12688487.v1 ^12^.

Data are available under the terms of the Creative Commons Zero "No rights reserved" data waiver (CC0 1.0 Public domain dedication).

## References

[1] Office of National Statistics: Overview of the UK population: August 2019.2019; Accessed 17 ^th^July 2020. Reference Source

[2] Public Health England: Older people’s hospital admissions in the last year of life.2020; Accessed 17 ^th^July 2020. Reference Source

[3] NHS Improvement: Guide to reducing long hospital stays.2018; Accessed 17 ^th^July 2020. Reference Source

[4] Humphries R Hall P Charles A: Social care for older people: Home truths. The King’s Fund and The Nuffield Trust.2016; Accessed 20th June 2020. Reference Source

[5] National Audit Office: Discharging older patients from hospital.2016; Accessed 20 ^th^June 2020. Reference Source

[6] Commission on the Future of Health and Social Care in England: A new settlement for health and social care: Final report. London: The King’s Fund; 202014. Accessed 18th June 2020. Reference Source

[7] NHS England: Five year Forward View.2014; Accessed 18 ^th^June 2020. Reference Source

[8] Charles A: Integrated care systems explained: making sense of systems, places and neighbourhoods. Accessed 18 ^th^June 2020. Reference Source

[9] Aromataris E Fernandez R Godfrey C: Chapter 10: Umbrella Reviews. In: Aromataris E, Munn Z (Editors). *JBI Manual for Evidence Synthesis.*JBI.2020; Accessed 10 ^th^June 2020. 10.46658/JBIMES-20-11

[10] Moher D Liberati A Tetzlaff J: Preferred Reporting Items for Systematic Reviews and Meta-Analyses: The PRISMA Statement. *PLoS Med.* 2009;6(7):e1000097. 10.1371/journal.pmed.1000097 19621072 PMC2707599

[11] Lunny C McKenzie JE McDonald S: Retrieval of overviews of systematic reviews in MEDLINE was improved by the development of an objectively derived and validated search strategy. *J Clin Epidemiol.* 2016;74:107–118. 10.1016/j.jclinepi.2015.12.002 26723872

[12] Dawson S Kunonga P Beyer F: Does health and social care provision for the community dwelling older population help to reduce unplanned secondary care, support timely discharge and improve patient well-being? A mixed method meta-review of systematic reviews. *figshare.*Journal contribution.2020. 10.6084/m9.figshare.12688487.v1 PMC8482050 34621521

[13] Centre for Reviews and Dissemination Database of Abstracts and Reviews of Effects CRD DARE. Accessed 29 ^th^April 2018. Reference Source

[14] Care Act 2014, Chapter 23. London: The Stationery Office. Accessed 28 ^th^June 2020. Reference Source

[15] Whiting P Savović J Higgins JP: ROBIS: A new tool to assess risk of bias in systematic reviews was developed. *J Clin Epidemiol.* 2016;69:225–234. 10.1016/j.jclinepi.2015.06.005 26092286 PMC4687950

[16] Lewin S Booth A Glenton C: Applying GRADE-CERQual to qualitative evidence synthesis findings: introduction to the series. *Implement Sci.* 2018;13(Suppl 1):2. 10.1186/s13012-017-0688-3 29384079 PMC5791040

[17] Graverholt B Forsetlund L Jamtvedt G: Reducing hospital admissions from nursing homes: a systematic review. *BMC Health Serv Res.* 2014;14:36. 10.1186/1472-6963-14-36 24456561 PMC3906881

[18] Philp I Mills KA Thanvi B: Reducing hospital bed use by frail older people: results from a systematic review of the literature. *Int J Integr Care.* 2013;13:e048. 10.5334/ijic.1148 24363636 PMC3860583

[19] Wong KC Wong FKY Yeung WF: The effect of complex interventions on supporting self-care among community-dwelling older adults: a systematic review and meta-analysis. *Age Ageing.* 2018;47(2):185–193. 10.1093/ageing/afx151 28927235

[20] Jonkman NH Schuurmans MJ Groenwold RHH: Identifying components of self-management interventions that improve health-related quality of life in chronically ill patients: Systematic review and meta-regression analysis. *Patient Educ Couns.* 2016;9(7):1087–1098. 10.1016/j.pec.2016.01.022 26856778

[21] Majothi S Jolly K Heneghan NR: Supported self-management for patients with COPD who have recently been discharged from hospital: a systematic review and meta-analysis. *Int J Chron Obstruct Pulmon Dis.* 2015;10:853–867. 10.2147/COPD.S74162 25995625 PMC4425235

[22] Jordan RE Majothi S Heneghan NR: Supported self-management for patients with moderate to severe chronic obstructive pulmonary disease (COPD): an evidence synthesis and economic analysis. *Health Technol Assess.* 2015;19(36):1–516. 10.3310/hta19360 25980984 PMC4781645

[23] Baker E Fatoye F: Clinical and cost effectiveness of nurse-led self-management interventions for patients with copd in primary care: A systematic review. *Int J Nurs Stud.* 2017;71:125–138. 10.1016/j.ijnurstu.2017.03.010 28399427

[24] Newham JJ Presseau J Heslop-Marshall K: Features of self-management interventions for people with COPD associated with improved health-related quality of life and reduced emergency department visits: a systematic review and meta-analysis. *Int J Chron Obstruct Pulmon Dis.* 2017;12:1705–1720. 10.2147/COPD.S133317 28652723 PMC5473493

[25] Harkness K Spaling MA Currie K: A systematic review of patient heart failure self-care strategies. *J Cardiovasc Nurs.* 2015;30(2):121–135. 10.1097/JCN.0000000000000118 24651683

[26] Lennon S McKenna S Jones F: Self-management programmes for people post stroke: a systematic review. *Clin Rehabil.* 2013;27(10):867–878. 10.1177/0269215513481045 23543340

[27] Pearce G Pinnock H Epiphaniou E: Experiences of Self-Management Support Following a Stroke: A Meta-Review of Qualitative Systematic Reviews. *PLoS One.* 2015;10(12):e0141803. 10.1371/journal.pone.0141803 26657458 PMC4682853

[28] Moore E Palmer T Newson R: Pulmonary Rehabilitation as a Mechanism to Reduce Hospitalizations for Acute Exacerbations of COPD: A Systematic Review and Meta-Analysis. *Chest.* 2016;150(4):837–859. 10.1016/j.chest.2016.05.038 27497743

[29] de Sousa Pinto JM Martín-Nogueras AM Morano MT: Chronic obstructive pulmonary disease patients' experience with pulmonary rehabilitation: a systematic review of qualitative research. *Chron Respir Dis.* 2013;10(3):141–157. 10.1177/1479972313493796 23897930

[30] Jenkins AR Gowler H Curtis F: Efficacy of supervised maintenance exercise following pulmonary rehabilitation on health care use: a systematic review and meta-analysis. *Int J Chron Obstruct Pulmon Dis.* 2018;13:257–273. 10.2147/COPD.S150650 29391784 PMC5768431

[31] Beauchamp MK Evans R Janaudis-Ferreira T: Systematic review of supervised exercise programs after pulmonary rehabilitation in individuals with COPD. *Chest.* 2013;144(4):1124–1133. 10.1378/chest.12-2421 23429931

[32] Borge CR Hagen KB Mengshoel AM: Effects of controlled breathing exercises and respiratory muscle training in people with chronic obstructive pulmonary disease: results from evaluating the quality of evidence in systematic reviews. *BMC Pulm Med.* 2014;14:184. 10.1186/1471-2466-14-184 25416306 PMC4258938

[33] Wu W Liu X Wang L: Effects of Tai Chi on exercise capacity and health-related quality of life in patients with chronic obstructive pulmonary disease: a systematic review and meta-analysis. *Int J Chron Obstruct Pulmon Dis.* 2014;9:1253–1263. 10.2147/COPD.S70862 25404855 PMC4230171

[34] Chen YM Li Y: Safety and efficacy of exercise training in elderly heart failure patients: a systematic review and meta-analysis. *Int J Clin Pract.* 2013;67(11):1192–1198. 10.1111/ijcp.12210 24165432

[35] Younge JO Gotink RA Baena CP: Mind-body practices for patients with cardiac disease: a systematic review and meta-analysis. *Eur J Prev Cardiol.* 2015;22(11):1385–1398. 10.1177/2047487314549927 25227551

[36] Li G Yuan H Zhang W: Effects of Tai Chi on health related quality of life in patients with chronic conditions: a systematic review of randomized controlled trials. *Complement Ther Med.* 2014;22(4):743–755. 10.1016/j.ctim.2014.06.003 25146080

[37] Stevens Z Barlow C Kendrick D: Effectiveness of general practice-based physical activity promotion for older adults: systematic review. *Prim Health Care Res Dev.* 2014;15(2):190–201. 10.1017/S1463423613000017 23506656

[38] Svensson HK Olsson LE Hansson T: The effects of person-centered or other supportive interventions in older women with osteoporotic vertebral compression fractures-a systematic review of the literature. *Osteoporos Int.* 2017;28(9):2521–2540. 10.1007/s00198-017-4099-8 28585054 PMC5550548

[39] Morilla-Herrera JC Garcia-Mayor S Martín-Santos FJ: A systematic review of the effectiveness and roles of advanced practice nursing in older people. *Int J Nurs Stud.* 2016;53:290–307. 10.1016/j.ijnurstu.2015.10.010 26542652

[40] Huntley AL Thomas R Mann M: Is case management effective in reducing the risk of unplanned hospital admissions for older people? A systematic review and meta-analysis. *Fam Pract.* 2013;30(3):266–275. 10.1093/fampra/cms081 23315222

[41] Gallagher C Elliott AD Wong CX: Integrated care in atrial fibrillation: a systematic review and meta-analysis. *Heart.* 2017;103(24):1947–1953. 10.1136/heartjnl-2016-310952 28490616

[42] Huntley AL Johnson R King A: Does case management for patients with heart failure based in the community reduce unplanned hospital admissions? A systematic review and meta-analysis. *BMJ Open.* 2016;6(5):e010933. 10.1136/bmjopen-2015-010933 27165648 PMC4874181

[43] King AJL Johnson R Cramer H: Community case management and unplanned hospital admissions in patients with heart failure: A systematic review and qualitative evidence synthesis. *J Adv Nurs.* 2018;74(7):1463–1473. 10.1111/jan.13559 29495081

[44] Tan SB Williams AF Kelly D: Effectiveness of multidisciplinary interventions to improve the quality of life for people with Parkinson's disease: a systematic review. *Int J Nurs Stud.* 2014;51(1):166–174. 10.1016/j.ijnurstu.2013.03.009 23611510

[45] Phelan EA Debnam KJ Anderson LA: A systematic review of intervention studies to prevent hospitalizations of community-dwelling older adults with dementia. *Med Care.* 2015;53(2):207–213. 10.1097/MLR.0000000000000294 25588136 PMC4310672

[46] Loh ZW Cheen MH Wee HL: Humanistic and economic outcomes of pharmacist-provided medication review in the community-dwelling elderly: A systematic review and meta-analysis. *J Clin Pharm Ther.* 2016;41(6):621–633. 10.1111/jcpt.12453 27696540

[47] Thomas R Huntley AL Mann M: Pharmacist-led interventions to reduce unplanned admissions for older people: a systematic review and meta-analysis of randomised controlled trials. *Age Ageing.* 2014;43(2):174–187. 10.1093/ageing/aft169 24196278

[48] Wallerstedt SM Kindblom JM Nylén K: Medication reviews for nursing home residents to reduce mortality and hospitalization: systematic review and meta-analysis. *Br J Clin Pharmacol.* 2014;78(3):488–497. 10.1111/bcp.12351 24548138 PMC4243900

[49] Cooper JA Cadogan CA Patterson SM: Interventions to improve the appropriate use of polypharmacy in older people: a Cochrane systematic review. *BMJ Open.* 2015;5(12):e009235. 10.1136/bmjopen-2015-009235 26656020 PMC4679890

[50] Hill-Taylor B Walsh KA Stewart S: Effectiveness of the STOPP/START (Screening Tool of Older Persons' potentially inappropriate Prescriptions/Screening Tool to Alert doctors to the Right Treatment) criteria: systematic review and meta-analysis of randomized controlled studies. *J Clin Pharm Ther.* 2016;41(2):158–169. 10.1111/jcpt.12372 26990017

[51] Bekkat-Berkani R Wilkinson T Buchy P: Seasonal influenza vaccination in patients with COPD: a systematic literature review. *BMC Pulm Med.* 2017;17(1):79. 10.1186/s12890-017-0420-8 28468650 PMC5415833

[52] Huntley AL Chalder M Shaw ARG: A systematic review to identify and assess the effectiveness of alternatives for people over the age of 65 who are at risk of potentially avoidable hospital admission. *BMJ Open.* 2017;7(7):e016236. 10.1136/bmjopen-2017-016236 28765132 PMC5642761

[53] Malik M Moore Z Patton D: The impact of geriatric focused nurse assessment and intervention in the emergency department: A systematic review. *Int Emerg Nurs.* 2018;37:52–60. 10.1016/j.ienj.2018.01.008 29429847

[54] Karam G Radden Z Berall LE: Efficacy of emergency department-based interventions designed to reduce repeat visits and other adverse outcomes for older patients after discharge: A systematic review. *Geriatr Gerontol Int.* 2015;15(9):1107–1117. 10.1111/ggi.12538 26171554 PMC5008161

[55] Jay S Whittaker P Mcintosh J: Can consultant geriatrician led comprehensive geriatric assessment in the emergency department reduce hospital admission rates? A systematic review. *Age Ageing.* 2017;46(3):366–372. 10.1093/ageing/afw231 27940568

[56] Leppin AL Gionfriddo MR Kessler M: Preventing 30-day hospital readmissions: a systematic review and meta-analysis of randomized trials. *JAMA Intern Med.* 2014;174(7):1095–1107. 10.1001/jamainternmed.2014.1608 24820131 PMC4249925

[57] Damery S Flanagan S Combes G: Does integrated care reduce hospital activity for patients with chronic diseases? An umbrella review of systematic reviews. *BMJ Open.* 2016;6(11):e011952. 10.1136/bmjopen-2016-011952 27872113 PMC5129137

[58] Martinez-González NA Berchtold P Ullman K: Integrated care programmes for adults with chronic conditions: a meta-review. *Int J Qual Health Care.* 2014;26(5):561–70. 10.1093/intqhc/mzu071 25108537 PMC4195469

[59] Zhu QM Liu J Hu HY: Effectiveness of nurse-led early discharge planning programmes for hospital inpatients with chronic disease or rehabilitation needs: a systematic review and meta-analysis. *J Clin Nurs.* 2015;24(19–20):2993–3005. 10.1111/jocn.12895 26095175

[60] Lowthian JA McGinnes RA Brand CA: Discharging older patients from the emergency department effectively: a systematic review and meta-analysis. *Age Ageing.* 2015;44(5):761–770. 10.1093/ageing/afv102 26265674

[61] Allen J Hutchinson AM Brown R: User Experience and Care Integration in Transitional Care for Older People From Hospital to Home: A Meta-Synthesis. *Qual Health Res.* 2017;27(1):24–36. 10.1177/1049732316658267 27469975

[62] Blakey EP Jackson D Walthall H: What is the experience of being readmitted to hospital for people 65 years and over? A review of the literature. *Contemp Nurse.* 2017;53(6):698–712. 10.1080/10376178.2018.1439395 29421948

[63] Ospina MB Mrklas K Deuchar L: A systematic review of the effectiveness of discharge care bundles for patients with COPD. *Thorax.* 2017;72(1):31–39. 10.1136/thoraxjnl-2016-208820 27613539

[64] Echevarria C Brewin K Horobin H: Early Supported Discharge/Hospital At Home For Acute Exacerbation of Chronic Obstructive Pulmonary Disease: A Review and Meta-Analysis [published correction appears in COPD. 2017 Dec;14(6):674]. *COPD.* 2016;13(4):523–533. 10.3109/15412555.2015.1067885 26854816

[65] Pandor A Gomersall T Stevens JW: Remote monitoring after recent hospital discharge in patients with heart failure: a systematic review and network meta-analysis. *Heart.* 2013;99(23):1717–1726. 10.1136/heartjnl-2013-303811 23680885

[66] Feltner C Jones CD Cené CW: Transitional care interventions to prevent readmissions for persons with heart failure: a systematic review and meta-analysis. *Ann Intern Med.* 2014;160(11):774–784. 10.7326/M14-0083 24862840

[67] Van Spall HGC Rahman T Mytton O: Comparative effectiveness of transitional care services in patients discharged from the hospital with heart failure: a systematic review and network meta-analysis. *Eur J Heart Fail.* 2017;19(11):1427–1443. 10.1002/ejhf.765 28233442

[68] Qaddoura A Yazdan-Ashoori P Kabali C: Efficacy of Hospital at Home in Patients with Heart Failure: A Systematic Review and Meta-Analysis. *PLoS One.* 2015;10(6):e0129282. 10.1371/journal.pone.0129282 26052944 PMC4460137

[69] Dickson K Sutcliffe K Rees R: Gaps in the evidence on improving social care outcomes: findings from a meta-review of systematic reviews. *Health Soc Care Community.* 2017;25(4):1287–1303. 10.1111/hsc.12300 26500053 PMC5484323

[70] de São José J Barros R Samitca S: Older persons' experiences and perspectives of receiving social care: a systematic review of the qualitative literature. *Health Soc Care Community.* 2016;24(1):1–11. 10.1111/hsc.12186 25660372

[71] Pettersson C Iwarsson S: Evidence-based interventions involving occupational therapists are needed in re-ablement for older community-living people: A systematic review. *Br J Occup Ther.* 2017;80(5):273–285. 10.1177/0308022617691537

[72] Tessier A Beaulieu MD Mcginn CA: Effectiveness of Reablement: A Systematic Review. Efficacité de l'autonomisation: une revue systématique. *Healthc Policy.* 2016;11(4):49–59. 10.12927/hcpol.2016.24594 27232236 PMC4872552

[73] Boniface G Mason M MacIntyre J: The Effectiveness of Local Authority Social Services’ Occupational Therapy for Older People in Great Britain: A Critical Literature Review. *Br J Occup Ther.* 2013;76(12):538–547. 10.4276/030802213X13861576675240

[74] Coll-Planas L Nyqvist F Puig T: Social capital interventions targeting older people and their impact on health: a systematic review. *J Epidemiol Community Health.* 2017;71(7):663–672. 10.1136/jech-2016-208131 27834223

[75] Leung P Orrell M Orgeta V: Social support group interventions in people with dementia and mild cognitive impairment: a systematic review of the literature. *Int J Geriatr Psychiatry.* 2015;30(1):1–9. 10.1002/gps.4166 24990344

[76] Cabrera E Sutcliffe C Verbeek H: Non-pharmacological interventions as a best practice strategy in people with dementia living in nursing homes. A systematic review. *Geriatr Med.* 2015;6(2):134–150. 10.1016/j.eurger.2014.06.003

[77] Folkerts AK Roheger M Franklin J: Cognitive interventions in patients with dementia living in long-term care facilities: Systematic review and meta-analysis. *Arch Gerontol Geriatr.* 2017;73:204–221. 10.1016/j.archger.2017.07.017 28843172

[78] Gardiner C Geldenhuys G Gott M: Interventions to reduce social isolation and loneliness among older people: an integrative review. *Health Soc Care Community.* 2018;26(2):147–157. 10.1111/hsc.12367 27413007

[79] Cohen-Mansfield J Perach R: Interventions for alleviating loneliness among older persons: a critical review. *Am J Health Promot.* 2015;29(3):e109–e125. 10.4278/ajhp.130418-LIT-182 24575725

[80] Poscia A Stojanovic J La Milia DI: Interventions targeting loneliness and social isolation among the older people: An update systematic review. *Exp Gerontol.* 2018;102:133–144. 10.1016/j.exger.2017.11.017 29199121

[81] Van Malderen L Mets T Gorus E: Interventions to enhance the Quality of Life of older people in residential long-term care: a systematic review. *Ageing Res Rev.* 2013;12(1):141–150. 10.1016/j.arr.2012.03.007 22504403

[82] Hagan R Manktelow R Taylor BJ: Reducing loneliness amongst older people: a systematic search and narrative review. *Aging Ment Health.* 2014;18(6):683–693. 10.1080/13607863.2013.875122 24437736

[83] Franck L Molyneux N Parkinson L: Systematic review of interventions addressing social isolation and depression in aged care clients. *Qual Life Res.* 2016;25(6): 1395–1407. 10.1007/s11136-015-1197-y 26646806

[84] Shvedko A Whittaker AC Thompson JL: Physical activity interventions for treatment of social isolation, loneliness or low social support in older adults: a systematic review and meta-analysis of randomised controlled trials. *Psychol Sport Exerc.* 2018;34:128–137. 10.1016/j.psychsport.2017.10.003

[85] Chen YR Schulz PJ: The Effect of Information Communication Technology Interventions on Reducing Social Isolation in the Elderly: A Systematic Review. *J Med Internet Res.* 2016;18(1):e18. 10.2196/jmir.4596 26822073 PMC4751336

[86] Kachouie R Sedighadeli S Khosla R: Socially assistive robots in elderly care: a mixed-method systematic literature review. *International Journal of Human-Computer Interaction.* 2014;30(5): 369–393. 10.1080/10447318.2013.873278

[87] Chipps J Jarvis MA Ramlall S: The effectiveness of e-Interventions on reducing social isolation in older persons: A systematic review of systematic reviews. *J Telemed Telecare.* 2017;23(10):817–827. 10.1177/1357633X17733773 28958209

[88] Macdonald GM: Using Systematic Reviews to Improve Social Care.Bristol: The Policy Press and London: Social Care Institute for Excellence. 2003. Reference Source

[89] Coulter A Collins A: Making shared decision-making a reality: no decision about me without me.The King's Fund, London. 2011. Reference Source

[90] Pope C van Royen P Baker R: Qualitative methods in research on healthcare quality. *Qual Saf Health Care.* 2002;11(2):148–152. 10.1136/qhc.11.2.148 12448807 PMC1743608

[91] National Institute for Clinical Excellence: Transition Between Inpatient Hospital Settings and Community or Care Home Settings for adults with social care needs. NICE guidelines [NG27].London. 2015. Reference Source

[92] Elliott JH Turner T Clavisi O: Living systematic reviews: an emerging opportunity to narrow the evidence-practice gap. *PLoS Med.* 2014;11(2):e1001603. 10.1371/journal.pmed.1001603 24558353 PMC3928029

[93] Agency for Healthcare Research and Quality: SRDR: Systematic Review Data Repository. 2013. Accessed 8 November 2013. Reference Source

