# Supplementary material for: Does health and social care provision for the community dwelling older population help to reduce unplanned secondary care, support timely discharge and improve patient well-being? A mixed method meta-review of systematic reviews
Source: F1000Res. 2020 Jul 31;9:857. [Version 1] doi: 10.12688/f1000research.25277.1 (PMC8482050; doi:10.12688/f1000research.25277.1)
Supplement: Peer review history

# Peer review: Reviewer response for version 1

**DOI:** 10.5256/f1000research.27895.r95251

**Published:** 2021-09-29

**Authors:** Johannes Siegrist

**Affiliations:** 1 Centre for Health and Society, Faculty of Medicine, Heinrich-Heine-University of Düsseldorf, Düsseldorf, Germany

This is a well-conducted meta-review on a complex topic with relevance to health care of older people. Methodologically, I do not see any need for change or additional analysis. In terms of content, the main finding indicates a continued poor emphasis on integrated analysis of health and social care (as well as a methodologically under-developed state of intervention research on effectiveness of social care). The conclusions of this review are justified, and they include some fruitful suggestions for further developments.

 If I miss something relevant in this paper, it concerns the lack of discussing the impact of a country's  health care system on innovations in integrating health and social care. For instance, what can the UK system learn from developments in other countries? Maybe authors are in a position to discuss this issue, given the accumulated evidence from their literature search.

Are the rationale for, and objectives of, the Systematic Review clearly stated?

Yes

Is the statistical analysis and its interpretation appropriate?

Yes

Are sufficient details of the methods and analysis provided to allow replication by others?

Yes

Are the conclusions drawn adequately supported by the results presented in the review?

Yes

Reviewer Expertise:

Medical sociology and social epidemiology

I confirm that I have read this submission and believe that I have an appropriate level of expertise to confirm that it is of an acceptable scientific standard.

# Peer review: Reviewer response for version 1

**DOI:** 10.5256/f1000research.27895.r68566

**Published:** 2020-08-27

**Authors:** Clarice Tang

**Affiliations:** 1 Department of Physiotherapy, Western Health, Footscray, Vic, Australia; 2 Western Sydney University, Penrith, NSW, Australia

This review of the systematic reviews has been conducted with rigour and is well written. It does also provide important information about the lack of evidence in health and social care interventions which is in-line with the current level of evidence around this literature (Poupard *et al. *2020). ^1^ 

 My only comment is to consider the potential lack of evidence in the lack of evidence in health and social care interventions may be due to other reasons apart from the lack of objective outcomes in social support which has not been highlighted in the discussion. Firstly, disease-specific management such as heart failure and COPD do have specific evidence-based clinical pathways which will likely result in a positive effect on medical management for these particular diseases as compared to older people with mixed conditions. Secondly, the type of care management differed greatly across the various studies. The length of interventions and time of implementation of care management may have resulted in a different outcome. Comments regarding if factors such as length of interventions and timing of interventions impacted on the final results will be helpful for the readers.

Are the rationale for, and objectives of, the Systematic Review clearly stated?

Yes

Is the statistical analysis and its interpretation appropriate?

Yes

Are sufficient details of the methods and analysis provided to allow replication by others?

Yes

Are the conclusions drawn adequately supported by the results presented in the review?

Yes

Reviewer Expertise:

Health service management, chronic disease, physical therapy

I confirm that I have read this submission and believe that I have an appropriate level of expertise to confirm that it is of an acceptable scientific standard.

## References

[1] : Community-based case management does not reduce hospital admissions for older people: a systematic review and meta-analysis.*Aust Health Rev*.2020;44(1) : 10.1071/AH1813583-9210.1071/AH1813530728095
